# Error Rates of Data Processing Methods in Clinical Research: A Systematic Review and Meta-Analysis of Manuscripts Identified Through PubMed

**DOI:** 10.21203/rs.3.rs-2386986/v2

**Published:** 2023-12-21

**Authors:** Maryam Y. Garza, Tremaine Williams, Songthip Ounpraseuth, Zhuopei Hu, Jeannette Lee, Jessica Snowden, Anita C. Walden, Alan E. Simon, Lori A. Devlin, Leslie W. Young, Meredith N. Zozus

**Affiliations:** 1 Department of Biomedical Informatics, University of Arkansas for Medical Sciences, Little Rock, Arkansas; 2 Department of Biostatistics, University of Arkansas for Medical Sciences, Little Rock, Arkansas; 3 Department of Pediatrics, University of Arkansas for Medical Sciences, Little Rock, Arkansas; 4 University of Colorado Denver, Anschutz Medical Campus, Denver, Colorado; 5 Environmental influences on Child Health Outcomes (ECHO) Program, National Institutes of Health (NIH), Rockville, Maryland*; 6 Department of Pediatrics, University of Louisville, Louisville, Kentucky; 7 Department of Pediatrics, The Larner College of Medicine at the University of Vermont, Burlington, Vermont; 8 University of Texas Health Science Center at San Antonio, Joe R. & Teresa Lozano Long School of Medicine, San Antonio, Texas

**Keywords:** medical record abstraction, data quality, clinical research, clinical data management, data collection

## Abstract

**Background:**

In clinical research, prevention of systematic and random errors of data collected is paramount to ensuring reproducibility of trial results and the safety and efficacy of the resulting interventions. Over the last 40 years, empirical assessments of data accuracy in clinical research have been reported in the literature. Although there have been reports of data error and discrepancy rates in clinical studies, there has been little systematic synthesis of these results. Further, although notable exceptions exist, little evidence exists regarding the relative accuracy of different data processing methods. We aim to address this gap by evaluating error rates for 4 data processing methods.

**Methods:**

A systematic review of the literature identified through PubMed was performed to identify studies that evaluated the quality of data obtained through data processing methods typically used in clinical trials: medical record abstraction (MRA), optical scanning, single-data entry, and double-data entry. Quantitative information on data accuracy was abstracted from the manuscripts and pooled. Meta-analysis of single proportions based on the Freeman-Tukey transformation method and the generalized linear mixed model approach were used to derive an overall estimate of error rates across data processing methods used in each study for comparison.

**Results:**

A total of 93 papers (published from 1978 to 2008) meeting our inclusion criteria were categorized according to their data processing methods. The accuracy associated with data processing methods varied widely, with error rates ranging from 2 errors per 10,000 fields to 2,784 errors per 10,000 fields. MRA was associated with both high and highly variable error rates, having a pooled error rate of 6.57% (95% CI: 5.51, 7.72). In comparison, the pooled error rates for optical scanning, single-data entry, and double-data entry methods were 0.74% (0.21, 1.60), 0.29% (0.24, 0.35) and 0.14% (0.08, 0.20), respectively.

**Conclusions:**

Data processing and cleaning methods may explain a significant amount of the variability in data accuracy. MRA error rates, for example, were high enough to impact decisions made using the data and could necessitate increases in sample sizes to preserve statistical power. Thus, the choice of data processing methods can likely impact process capability and, ultimately, the validity of trial results.

## Background

In regulated clinical trials, investigators must rely on research data acquired to (1) ensure the safety and efficacy of medical treatments (to protect research participants and the general population at large), and (2) ensure the reliability and reproducibility of study results. High quality data provide the foundation from which study conclusions may be drawn,^1^ and, in contrast, poor data quality threatens the validity and generalizability of study findings.^1,2^ In general, *quality* refers to “a product or service free of deficiencies”^1,3^ – some experts also using terms like “fitness for use”^4^ and “conformance to requirements.”^5^ Within the context of clinical research and the practice of clinical data management, the Institute of Medicine defines *data quality* as data that “support the same conclusions as error free data.”^6^ There are several attributes tied to quality, but, for this project, we focused primarily on *data accuracy* – data that accurately represent data points collected directly from study participants.^1^

Authors in the clinical research arena lament the scarcity of published information regarding data quality.^6–18^ While many authors point out that conclusions drawn from studies depend on data quality (and the underlying data collection and management methods), others consider the associated tasks clerical or even unnecessary.^19–22^ This perception has resulted in minimal investigation and a small number of publications on the topic of data collection and management compared with other areas of clinical research and informatics methodology. With the current rapid influx of new technology into clinical research – starting with electronic data capture (EDC) and clinical trial management systems (CTMSs) shortly after the turn of the century, and followed by electronic patient reported outcomes (ePRO) systems, mobile health (mHealth), a myriad of digital health technologies (DHTs), and direct electronic health record-to-electronic case report form (EHR-to-eCRF) tools – understanding the quality of data from different available capture and processing methods has become even more important.^23,24^ Many unresolved issues exist with respect to data quality in clinical research, including a thorough understanding of the accuracy and variability of current data processing methods^24–28^ – a primary objective of this manuscript. A thorough review and synthesis of the relevant published literature is an initial step in providing guidance to investigators and clinical research teams. Accordingly, we aimed to address this gap through the systematic review and meta-analysis described in this manuscript.

Common options in data processing methods identified in the literature include: (1) chart review and abstraction versus direct electronic acquisition from electronic medical records (i.e., both types of medical record abstraction, or MRA); (2) use of vended or commercial data collection systems by local healthcare facilities (e.g., data entry and cleaning in local systems versus web-based data entry and cleaning in a centrally hosted system); (3) use of paper data collection forms with central processing versus local processing with data transfer to a central coordinating center; and (4) single- versus double-data entry (with or without programmed edit checks). Data cleaning methods also vary greatly, from use of reports to identify irregularities in the data, to on-screen checks (OSCs) during data entry (e.g., programmed edit checks), to post-entry batch data processing. We define the 4 major processing methods considered in this review (MRA, optical scanning, single-data entry, and double-data entry with or without programmed edit checks) in Table 1.

Complicating comparisons of different data processing methods are the significant variability in quantitative methods for assessing data accuracy across clinical research and other secondary data uses.^1,29,30^ Data accuracy has often been measured in terms of database error rates, although, registries commonly assess percent completeness as well. To standardize, the Society for Clinical Data Management’s (SCDM) Good Clinical Data Management Practices (GCDMP) document has defined the *error rate* as the “number of errors divided by the number of data values inspected.”^1,31^

As described in the GCDMP,^1^ there are significant differences in the way errors and values are inspected and counted across different clinical research studies, even across those conducted by the same institution. Based on these counting differences, the error rates obtained can differ by a factor of 2 or more.^1,30^ In addition, differences in how error rates are reported (e.g., as raw counts, errors per record, errors per fields inspected, or errors per 10,000 fields), necessitate scaling and normalization of the values reported in the literature before comparisons can be made. Due to variability in counting, such comparisons may still not be meaningful. Here, we undertook a systematic review of the relevant literature identified through PubMed to characterize data collection and processing methods utilized in clinical studies and registries. Additionally, we conducted a meta-analysis to calculate and compare error rates across the various data processing methods described.

## Methods

### Literature Review

A PubMed search on the Medical Subject Heading (MeSH) terms “data quality” AND (registry OR “clinical research” OR “clinical trial”) through 2008 was conducted to identify relevant citations (see Additional File 1, Appendix A, Item A1 for the full PubMed Search Strategy and Table A2 for the PRISMA Checklist). Once an initial list of manuscripts was generated via PubMed, duplicates were excluded. The abstracts of the de-duplicated set of citations were screened for relevance against the eligibility criteria and those not meeting the criteria were also excluded. A search using PubMed related links and secondary and tertiary references was then conducted to identify additional manuscripts. The full-text of included manuscripts was reviewed against the eligibility criteria to generate the final set of manuscripts for inclusion in analysis (see Additional File 1, Appendix A, Reference List A3 and Table A4).

### Criteria for Manuscript Inclusion

The goal of this search was to identify quantitative reports of data quality in clinical studies, and the search terms and logic were selected to optimize that. If we consider this review in terms of the commonly used Patient/Population, Intervention, Comparison, Outcomes (PICO) framework^32^ for clinical searches, we can break down our search as follows. The population of interest was “clinical studies” – more specifically, “registries” or “clinical research” or “clinical trials” that relied on secondary use of healthcare data. The intervention of interest was “data processing methods” – in other words, activities that were carried out during the study to acquire, process, and/or manage the data of interest. As our research question was one of characterization, we did not look for papers reporting methodological comparisons. With respect to outcome, we required a quantitative reports of data quality such that we could calculate an error rate on the level of data values in error divided by the number of data values inspected.

Manuscripts were included in the analysis if: (1) they were published in peer reviewed journals indexed for retrieval or referenced by such and were obtainable; (2) they had a focus on secondary data use of healthcare data (e.g., clinical research, quality improvement, surveillance, research registries); (3) the database error rate was presented or resolvable (e.g., via number of errors identified and number of fields inspected, or contained sufficient information to calculate); (4) they described how the data were processed (e.g., MRA, optical scanning, single- or double-data entry); (5) they were written in the English language; and (6) the manuscript was the primary source for the error rate. Manuscripts not meeting 1 or more of these inclusion criteria were excluded.

### Information Gathered from Manuscripts

Three types of data were collected from each manuscript: (1) information about how data were processed; (2) information about how data quality was measured; and (3) the number of errors and number of fields inspected. Concepts of interest of the data processing and quality measurement methods reported were noted as each manuscript was read. Prior to quantitative data analysis, factors identified from items (1) and (2) were developed in a qualitative, iterative manner during the review of the manuscripts. As such, concepts of interest, such OSCs versus batch data discrepancy identification were added to the data collection form as they were identified, and previously reviewed manuscripts were re-reviewed for presence of the newly identified concepts of interest. Natural groupings were organized into categories. These categories were later explored in the analysis to ascertain which (if any) of the factors might affect data quality.

The following parameters were also collected, but were considered supplemental: data cleaning method (i.e., batch data cleaning), location of data processing (central data center vs. local healthcare facility), gold standard used, and scope of method of comparison.

Quantitative data accuracy information including the number of errors identified and the number of fields inspected was abstracted from the manuscripts. Manuscripts were categorized by type of secondary data use, data processing method, and data accuracy assessment. Information on the number of errors identified and the number of fields inspected was collected for each manuscript. We abstracted the number of errors reported and the total number of data fields (values) inspected. The number of errors and number of fields inspected were used to calculate normalized error rates (number of errors per 10,000 fields) based on the recommendations in the GCDMP.^1^ In cases where the authors presented only normalized error rates, such as errors per 10,000 fields, the normalized denominator was assumed for the total number of fields inspected. For example, if the normalized error rate presented was 100 per 10,000 fields, we took 100 to be the total number of errors (numerator) and 10,000 to be the total number of fields (denominator). Where error rates for more than 1 database were provided in a manuscript, each individual assessment was included in this analysis. Where error rates for multiple data processing steps were provided, we included each.

For consistency, 1 rater was used to abstract the error rate information from the manuscripts. A sample of the manuscripts included in the analysis, comprising 10% of the total (standard for the domain), was re-evaluated by the primary rater following the initial abstraction to assess reliability. For the sample, the time between the initial and intra-rater reliability review was at least 1 year. Intra-rater reliability, calculated as percent difference, was used to gauge reliability of the data. In addition, a second rater reviewed the same intra-rater reliability sample.

### Statistical Analysis

Meta-analysis of single proportions^33,34^ based on the Freeman-Tukey transformation method^35^ and the generalized linear mixed model approach^36^ of studies from the literature were used to derive an overall estimate of error rates across data processing methods for comparison. We also performed subgroup analyses where the data allowed. All statistical tests were performed at a two-sided significant level of 0.05, and all analyses were carried out using the R package ‘metafor’ and ‘meta’.^37,38^ For each of the data processing methods, we used an inverse variance weighted meta-analytical method with Freeman-Tukey transformation^35^ to calculate the pooled effect size and corresponding 95% confidence interval (CI). In the analysis, records with studentized residuals greater than an absolute value of 3 were considered outliers and subsequently removed. The degree of heterogeneity between studies were examined based on the Q-statistic and Higgins and Thompson’s I^2^ statistic. The I^2^ statistic can be interpreted approximately as ≤ 25%, indicating low heterogeneity; 25% to 75% indicating moderate heterogeneity; and > 75%, indicating considerable heterogeneity.^39^ The Q-statistic is typically underpowered for detecting true heterogeneity when the number of studies is small; therefore, we pooled data using a random effects model. The inter-study variance was evaluated by computing tau-squared (τ^2^), which provides the estimated standard deviation of the underlying effects across studies. Finally, to evaluate the consistency of our study, a sensitivity analysis was conducted using a leave-one-out model.^40^ Also, a meta-regression with mixed-effect model with Freeman-Tukey transformation was implemented to compare the pooled effect among data processing methods.

## Results

### Manuscripts Included for Analysis

**Figure 1.**
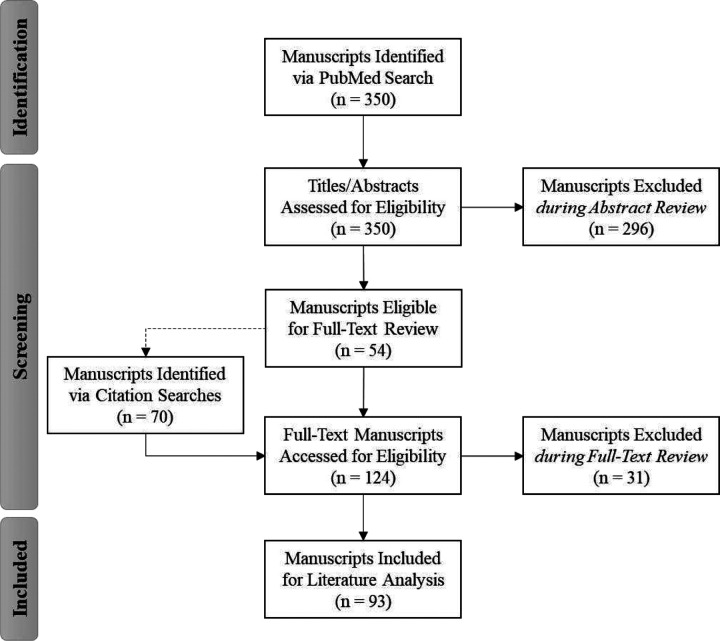
PRISMA Diagram: Identification of Data Quality Literature for Pooled Analysis

An initial search of the literature identified 350 citations. After excluding duplicates and performing the initial screen of abstracts, 54 manuscripts remained. A search using PubMed related links and secondary and tertiary references identified an additional 70 manuscripts, yielding 124 manuscripts for full-text review. Through the full-text review, we identified the final set of 93 manuscripts (see Additional File 1, Appendix A, Reference List A3 and Table A4), which were included in the pooled literature analysis (Figure 1).

Four manuscripts^41–44^ presented only normalized error rates as errors per 10,000 fields; for these, the denominator (10,000) was assumed for the total number of fields inspected. Each manuscript described a data quality assessment of 1 or more databases. Likewise, in some manuscripts, error rates were reported for more than 1 process step; for example, medical record-to-CRF or source-to-CRF, CRF-to-first entry, first entry-to-second entry, or CRF-to-clean file. A total of 22 manuscripts reported results for more than 1 processing step or database,^14,29,41,43,45–62^ providing a total of 124 data points normalized as number of errors per 10,000 fields and demonstrating increasing dispersion over time of the health-related research literature with respect to data accuracy. The data processing methods, as reported in the literature, were not mutually exclusive; thus, some articles appear in more than 1 category (see Additional File 1, Appendix A, Table A5).

### Meta-Analysis

During the meta-analysis, 9 records with absolute studentized residuals values greater than 3 were identified as outliers and, consequently, excluded from the analysis. Thus, 84 manuscripts remained, which were categorized by data processing method and were included in the final analysis. Database error rates ranged from 2 – 2,784 errors per 10,000 fields (having excluded outliers) across 4 data processing methods: MRA, optical scanning, single-data entry, and double-data entry. This 3 orders-of-magnitude range necessitated a logarithmic display. There appeared to be no pattern in the year-to-year reporting. The data processing method with the highest error rates was MRA, having a pooled error rate of 6.57% (95% CI: 5.51, 7.72) (Table 2). The 3 other processing methods (optical scanning, single-data entry, and double-data entry) had much lower pooled error rates at 0.74% (0.21, 1.60), 0.29% (0.24, 0.35) and 0.14% (0.08, 0.20), respectively (Table 2). Heterogeneity was observed in all 4 data processing methods (see Additional File 2, Appendix B, Figures B1–B4). The sensitivity analysis did not indicate the extreme influence of any particular study (see Additional File 3, Appendix C, Tables C1–C4).

### Subgroup Analysis

In exploring subgroups of the 4 main data processing methods, there is insufficient information in the literature about the MRA methods employed to further investigate possible causes for the variability in a subgroup analysis. Similarly, there were too few optical methods data points to support a subgroup analysis. For single- and double-data entry, a review of the literature surfaced different variations on key entry, including single-data entry (1 person enters the data), single-data entry with on-screen data checks (1 person enters the data within a system employing programmatic, OSCs), and double-data entry (2 people independently enter data with a third, independent adjudicator to review and resolve discrepancies). Further variations on single-data entry found in the literature included, use of batch data cleaning, and the location of data processing. These results are provided in Additional File 4, Appendix D, Table D1. Due to the importance of this particular model, manuscripts reporting data accuracy from similar data processing configurations (e.g., central versus distributed data processing in the presence of OSC), were examined (see Additional File 4, Appendix D, Table D2). Sixty-eight studies (across 49 manuscripts) versus 49 studies (across 39 manuscripts) reported central versus distributed processing; while 7 studies (across 5 manuscripts) did not report the location of data processing (noted in Table A4, see Additional File 1, Appendix A).

The intra-rater reliability for number of errors, number of fields, and error rate were 85%, 97%, and 86%, respectively. In addition, a second rater reviewed the same intra-rater reliability sample, with comparable results. In light of the underlying variability in the data, the variability in error rate calculation methods currently in use, and the aims of this study, these were considered reasonable. In addition, they were comparable to those in a similar review paper of errors in EHRs.^63^

## Discussion

This study calculated and compared error rates across the various data processing methods described in the literature. The results indicated that the accuracy associated with data processing methods varied widely. Error rates ranged from 2 to 2,784 errors per 10,000 fields within the 4 most common data processing methods, strengthening our understanding of the influence of data processing and cleaning methods on data accuracy.

### Medical Record Abstraction

Ordered by the mean, MRA was associated with the highest error rate. Importantly, abstraction was also associated with significant variability. Notably, the error rates reported for MRA methods span 3 orders of magnitude, with error rates ranging from 70 to 2,784 errors per 10,000 fields. These results support claims that MRA, which remains the dominant method of data collection in retrospective and prospective research, is the most significant source of error across data processing methods.^13,64^

### Optical Scanning

Although optical scanning methods such as OCR and OMR have been touted as a faster, higher-quality or less resource-intensive substitute for manual data entry,^19,54,65–71^ others have reported error rates with optical methods that were 3 times higher than manual keyboard data entry.^72^ Based on the pooled literature, we found optical scanning error rates ranged from 2 to 358 errors per 10,000 fields. Optical methods were associated with a variability of 2 orders of magnitude in accuracy. Such variability may be influenced by: (1) the presence and type of data cleaning employed in processing the optical scans; (2) use of post-entry visual verification or pre-entry manual review; (3) training of form completers on handwriting; (4) differences in form compatibility with the software; (5) software configuration (e.g., recognition engine); and (6) variations in data quality assessment methods. In particular, based on the available error in human inspection in other disciplines ranging from 16.4% to 30.0%,^73–77^ using manual visual verification is likely less effective than OSCs.

### Single- vs. Double-Data Entry

Overall, single-entry error rates ranged from 4 to 650 errors per 10,000 fields, and double-entry error rates ranged from 4 to 33 errors per 10,000 fields. Great variability was observed between different sub-types of single-data entry, which provides a plausible explanation for the high level of variability observed in single-data entry as a whole. This is an important finding because large amounts of data are collected through single-data entry from research sites via web-based systems, including entry of abstracted data into web-based systems, clinicians entering data in EHRs, and data collected directly from patients via hand-held devices. Due to the problem of “alert fatigue,” however, OSCs may not be feasible in EHRs, where clinical alerts will often be a higher priority. The question of alert fatigue in these systems is an important topic for further research.

### Measuring Data Accuracy

Claiming to have measured data accuracy (or error) is a statement implying that the measurer has compared the data to something, identified differences, and, in the case of a difference, was able to discern whether the data value from the assessed dataset was in error or not. In other words, a gold standard exists. In addition to aforementioned differences in counting errors and data values inspected, there was also variability in the literature with respect to the comparison made to measure data accuracy. In some cases, the comparator was the medical record; in other cases, it was an upstream recording of the data; in other cases, it was another dataset supposed to contain the same observations on the same individuals; and still in other cases, it was independent collection of the same information, such as a repeat interview or test. As evidenced by the literature and practice standard^78^ the error rate has historically been the accuracy metric used. However, use of sensitivity and specificity have been recommended in draft regulatory guidance as the preferred measures of accuracy in the case of EHR and claims real-world data (RWD).^79^ Sensitivity and specificity are preferred over overall accuracy or error rates because they are not dependent on prevalence.^80^ These measures were not often used in the included manuscripts, probably due to a long history of using accuracy (the sum of true positives and true negatives divided by the total number of data values inspected) or error rate (the sum of false positives and false negatives divided by the same denominator) metrics. Where a gold standard is not available, errors cannot be determined in the case of a difference, and the difference or discrepancy rate is tallied instead. In this case, only measures of agreement such as inter-rater reliability and chance-adjusted agreement are appropriate. There are many such measures.^81^ These measures, along with measures of agreement, were far more commonly in the included manuscripts than sensitivity and specificity. It is important to note that, while agreement may correlate with accuracy, agreement measures are not measures of data accuracy and, in many cases, may differ substantially from measures of accuracy.

As web-based EDC leads as the predominant method of future clinical research data collection, we anticipate heavier reliance on programmed edit checks to reduce error rates. Additionally, the role and process of programmed edit checks could serve as a model for addressing data quality checks of error rates within the more automated, standards-based processes of future data exchange, such as direct EHR-to-eCRF methods using the Health Level Seven (HL7^®^) Fast Healthcare Interoperability Resources (FHIR^®^) standard.^82–87^

### Limitations

This study was a secondary, pooled analysis of database error rates in the published literature. Although it constitutes an important contribution in synthesizing the very fragmented historical literature, there are significant and inherent limitations. Very few of the included papers were controlled studies. Most of the included manuscripts merely stated the observed error rate and described data handling methods as part of reporting research results from a clinical study. With the exception of 8 included manuscripts (manuscripts 15, 36, 39, 42, 45, 71, 88, 92 from Appendix A, Table A4), the included studies were observational in nature (a “one shot” design) and lacked a comparator, i.e., “low quality evidence”. It is ironic that the level of rigor expected of evidence is not expected of the methods used to generate it. The risk of bias in included studies is significant. However, we do not claim cause and report only associations and provide multiple possible explanations for them, which may encompass domains of bias. The ROBINS-I (Risk Of Bias In Non-randomized Studies - of Interventions) tool enumerates 9 domains of bias: confounding, selection of participants into the study, classification of intervention, deviations from intended interventions, missing data, measurement of outcomes, and selection of the reported result.^88^ For this research, we acknowledge that confounding could be present in any of the non-randomized included studies; for example, those reporting use of programmatic data quality checks may be more quality-conscious, or generally more careful. In general, reports tended to random sampling or census, obviating the second domain of bias.

A lack of standard terminology for data processing methods potentially affected this analysis through the high likelihood that relevant manuscripts were not identified or that descriptions in existing manuscripts were misinterpreted, i.e., bias from misclassification of the intervention. Though misclassification of the intervention (data processing method) was done systematically by the research team, the descriptions in the included studies themselves may be a source of bias.

As a secondary analysis, this work relies on data that were collected for other purposes. Although we used error and field counts reported in the literature, prior work has shown that even these have significant variability.^1,30^ For example, some may count dates as discrepant if there is not an exact match, while others may allow a window of several days; field counts may exclude null fields, or include fields entered once and propagated to multiple places.^89^ There likely is a bias toward counting rules that yield a larger denominator and smaller numerator. These represent a potential bias in measurement of outcome, and in handling missing data. Selection of the reported result or reporting bias is likely to be significant with reports tending toward those with lower error rates. Though the latter would have impacted use of our results as an acceptance level, it would not have impacted the comparisons between error rates and data processing methods because all included studies were equally subject to reporting bias. Taken together, the risks of bias in included studies would tend toward lower reported error rates and less difference between data processing methods, since the ideal in all cases is low error rates.

As with any literature review, there is the possibility that we may have missed relevant manuscripts in our search. Further, while the search, screening, and abstraction of information from the manuscripts was systematic, the search was only executed in PubMed. Other databases, such as EMBASE, were not searched; thus, manuscripts indexed in other databases were not included. Therefore, our results should only be considered representative of the biomedical literature searchable through PubMed.

Most of the manuscripts in our review were from academic organizations and government or foundation-funded endeavors that employ different data collection and management methodologies. Although over the time span of the literature we reviewed, those methods have tended to converge, our results may be less applicable to industry funded studies. Though our results are relevant to EDC data collection and cleaning processes, having to exclude the EDC (no manuscripts past the year 2008) literature from this review is a limitation. Authors did not consistently report the processes undertaken for collection and processing, nor did they include the error rate. For example, as reported in Nahm and colleagues in 2008,^23^ some sites used paper worksheets to record data abstracted from medical records, while others charted source data directly in such worksheets, versus others that abstracted directly from the medical record into the EDC system without a paper intermediary. Because these aspects often could not be resolved in published manuscripts, the review was truncated to account for the onset of EDC adoption, with the latest included manuscript published in 2008.

Exclusion of the EDC literature would have impacted applicability today of the MRA error rate results most significantly. For abstracted data recorded directly into EDC, use of on-screen checks would likely reduce the error rate. The lack of data accuracy quantification with EDC processes reported by Zozus and colleagues (2020) was evident in 2 of the 12 reports^23,90^ of data quality measured for EDC processes, reporting an error rate that would have met inclusion criteria for this study. A recent review summarized the EDC data quality literature and found only similarly absent or altogether lacking descriptions of data collection and processing methods accompanying reports of research results remains a serious omission.^24,91^

### Future Direction

As data (increasingly captured electronically) are used to support clinical research, the effects of data quality on decision-making need thorough exploration. Potential effects of system usability and data processing methods on data quality should also be characterized to guide data management and planning choices. In particular, the 2018 revision of Good Clinical Practices (GCP) calls for risk-based prioritization of study activities that focus resources on activities that impact human safety and research results.^92^ Use of the word *ensure* rather than *assure* in the guidance strongly suggests that quality management systems be in place to prospectively design capable processes and to control error rates within acceptable limits. We found very few reports of prospective prediction of process capability or of implementation of process control for the data error rate. Quality management system (QMS) design and implementation with respect to data accuracy remains an area for further exploration. The variability and the magnitude of error rates reported in the literature should encourage quantitative evaluation of the impact of new technology and processes on data accuracy and subsequent decisions regarding whether the accuracy of the data is acceptable for the intended use.

## Conclusion

Based on the pooled analysis of error rates from the published literature, we conclude that data processing and cleaning methods used in clinical trials research may explain a significant amount of the variability in data accuracy. For example, MRA error rates were associated with the highest and most variable compared to other data collection and processing methods, and that the observed error rates in the top quartile (904 to 2,784 errors per 10,000 fields) were high enough to potentially impact the results and interpretation of many clinical studies. In general, error rates reported in the literature were well within ranges that could necessitate increases in sample sizes from 20% or more in order to preserve statistical power for a given study design.^93,94^ Data errors also been shown to change p values^95^ and attenuate correlation coefficients to the null hypothesis;^96–98^ in other words, a given clinical trial may fail to reject the null hypothesis because of data errors rather than because of a genuine lack of effect for the experimental therapy.^99^ In the presence of large data error rates, a researcher must then choose to either (1) accept unquantifiable loss of statistical power and risk failure to reject the null hypothesis due to data error; or (2) measure the error rate and increase the sample size to maintain the original desired power.^89,94,98^ The adverse impact of data errors has also been demonstrated in registries and performance measurements,^55,99–103^ as has failure to report data.^104^ Thus, the choice of data processing methods can likely impact process capability and, ultimately, the validity of trial results. Our findings suggest that reporting the results of a clinical study without specifying (1) the error rate, (2) the uncertainty in the error rate, and (3) the method used to measure the error rate limits the ability to interpret study findings.

While such results in aggregate are shocking, we do not present them to incite panic or cast doubt upon clinical research results. Other factors that are not assessable here, such as variables in which the errors occurred, and statistical methods used to take the measurement error into account, are necessary for such assessments. We applaud the authors of the reviewed papers for their rigor and forthrightness in assessing error; measurement is the first step in management. We hope that our analysis makes a strong and convincing argument for the measurement and publication of data accuracy in clinical research.

## Figures and Tables

**Table 1.** Definitions of Data Processing Methods in Clinical Research

Data Processing Method	Definition
Medical Record Abstraction (MRA)	A data processing method that involves the review and abstraction of data from patient records, often referred to as *chart review* or *chart abstraction*. Traditional MRA is a manual process, which may or may not involve paper forms.
Optical Scanning (OMR)*	A data processing method that relies on software packages to “recognize characters from paper forms or faxed images, and these data are placed directly into the database.”^1^ Examples include *optical character recognition* (OCR) and *optical mark recognition* (OMR).
Single-Data Entry (SDE)*	With respect to classification of data processing in the included manuscripts, single-data entry involves 1 person who enters data from a structured form into the study data capture system. SDE can be implemented with and without programmed edit checks (or OSCs)*.
Double-Data Entry (DDE)*	Double-data entry involves 2 people (e.g., clinical research coordinator, data entry personnel) who independently enter data from a structured form to the study data capture system with a third, independent adjudicator to review and resolve any discrepancies. DDE can be implemented with and without programmed edit checks (or OSCs)*.

* Note. Hereinafter, we use a single acronym, OMR, to encompass all optical scanning methods discussed in the literature. Both single- and double-data entry methods can be conducted with or without programmed edit checks. Programmed edit checks – also referred to as discrepancy checks, edit checks, OSCs, or query rules – are electronic data quality checks that are programmed into the study data collection system and are triggered by data entry, either in real-time as data is entered or in batches.

**Table 2.** Pooled Effect Size of Error Rates (%) by Data Processing Method

Data Processing Method	Range	Pooled Proportion (95% CI)	p-value*
Abstraction (MRA)*	70 – 2,784	6.57 (5.51, 7.72)	Ref
Optical Scanning (OMR)	2 – 358	0.74 (0.21, 1.60)	<0.0001
Single-Data Entry (SDE)	4 – 650	0.29 (0.24, 0.35)	<0.0001
Double-Data Entry (DDE)	4 – 33	0.14 (0.08, 0.20)	<0.0001

* Note. Ref: Reference. p-values were calculated through meta-regression. Source-todatabase and source-to-CRF (case report form) were combined into a single category labeled abstraction, or MRA, based on: (1) some of the manuscripts reported error rates for abstraction directly to an electronic data collection form; i.e., no separate data entry step, and (2) the central tendency and dispersion of the 2 processes being similar.

## Data Availability

The dataset(s) supporting the conclusions of this manuscript is(are) included within the manuscript (and its additional file(s)).

## List of Terms & Abbreviations

EDC: Electronic Data Capture
CTMS: Clinical Trial Management System
ePRO: Electronic Patient Reported Outcomes
mHealth: Mobile Health
DHT: Digital Health Technology
EHR: Electronic Health Record
CRF: Case Report Form
eCRF: Electronic Case Report Form
MRA: Medical Record Abstraction: A data processing method that involves the review and abstraction of data from patient records, often referred to as *chart review* or *chart abstraction*. Traditional MRA is a manual process, which may or may not involve paper forms.
OSC: On-screen checks (i.e., programmed edit checks)
Optical Scanning: A data processing method used in clinical research that relies on software packages to “recognize characters from paper forms or faxed images, and these data are placed directly into the database.”^1^
OCR: Optical Character Recognition; an example of optical scanning
OMR: Optical Mark Recognition; an example of optical scanning
SDE: Single-Data Entry: With respect to classification of data processing in the included manuscripts, single-data entry involves 1 person who enters data from a structured form into the study data capture system. SDE can be implemented with and without programmed edit checks (or OSCs). When on-screen checks are employed, a series of programmatic edit checks are actively running during data entry and will “fire” when a discrepancy is identified during data entry. The data entry person is then able to review and address discrepancies during data entry.
Programmed Edit Checks: A data processing method during which electronic data quality checks are programmed into the study data collection system and are triggered by data entry, either in real-time as data is entered field-by-field or upon the form being saved, or in batch based on some pre-determined criteria. Programmed Edit Checks are also referred to as *Discrepancy Checks*, *Edit Checks*, *On-Screen Checks*, or *Query Rules*.
DDE: Double-Data Entry: Double-data entry involves 2 people (e.g., clinical research coordinator, data entry personnel) who independently enter data from a structured form to the study data capture system with a third, independent adjudicator to review and resolve any discrepancies. DDE can be implemented with and without programmed edit checks (or OSCs)*.
SCDM: Society for Clinical Data Management
GCDMP: Good Clinical Data Management Practices
MeSH: Medical Subject Heading
PICO: Patient/Population, Intervention, Comparison, Outcomes
CI: Confidence Interval
RWD: Real-World Data
HL7^®^: Health Level Seven
FHIR^®^: Fast Healthcare Interoperability Resources^87^
ROBINS-I: Risk Of Bias In Non-randomized Studies - of Interventions
GCP: Good Clinical Practice
QMS: Quality Management System
